# Behandlungsergebnisse nach vaskulärem Extremitätentrauma – erste Ergebnisse des vaskulären Traumaregisters Augsburg (VascTR-Aux)

**DOI:** 10.1007/s00104-025-02317-9

**Published:** 2025-06-05

**Authors:** Tobias Dominik Warm, Yaser Souri, Alexander Hyhlik-Dürr, Yvonne N. Goßlau

**Affiliations:** Klinik für Gefäßchirurgie und endovaskuläre Chirurgie, Medizinische Fakultät, Universität Augsburg, Stenglinstr. 2, 86156 Augsburg, Deutschland

**Keywords:** Vaskuläres Traumaregister, Extremitätenverletzungen, Postoperative Gerinnungshemmung, Gefäßverletzung, Polytrauma, Vascular trauma registry, Extremity injuries, Postoperative anticoagulation, Vascular injury, Polytrauma

## Abstract

**Hintergrund:**

Am Universitätsklinikum Augsburg wird ein prospektives vaskuläres Traumaregister (VascTR-Aux) geführt. Diese erste Auswertung der Registerdaten verfolgt das Ziel, die Behandlungsergebnisse nach vaskulären Extremitätenverletzungen zum Zeitpunkt der stationären Entlassung darzustellen.

**Material und Methoden:**

Von 01.01.2016 bis 31.03.2024 wurden in das VascTR-Aux 155 Daten von Verletzten eingeschlossen. Extremitätengefäße waren bei 83 Patientinnen und Patienten betroffen.

**Ergebnisse:**

Von den 83 eingeschlossenen Verletzten waren 62 männlich. Im Mittel betrug das Alter 37 Jahre.

Es lag in 28 Fällen eine Gefäßverletzung an der oberen und in 55 Fällen an der unteren Extremität vor. In 14 Fällen handelte es sich um Schwerverletzte. Klinisch präsentierten sich 29 Blutungen, 32 Ischämien und 14 Kombinationen aus beiden Entitäten. Sieben Fälle verliefen asymptomatisch. Die Therapie erfolgte offen chirurgisch (*n* = 51), als Hybridprozedur (*n* = 7) und endovaskulär (*n* = 8). In 76 Fällen war ein Funktionserhalt der Extremität möglich. In 6 Fällen war eine Major- und in 2 Fällen eine Minoramputation notwendig. Die mittlere Verweildauer lag bei 23 Tagen.

**Diskussion:**

Die initiale Therapie erfolgt häufig interdisziplinär, wohingegen die stationäre Weiterbetreuung aufgrund der behandlungsbedürftigen Begleitverletzungen oft bei anderen Fachabteilungen als der Gefäßchirurgie liegt. Das gefäßchirurgische Procedere bei Therapie und Nachsorge muss bei Gefäßverletzungen individuell besprochen und kommuniziert sowie eine gefäßchirurgische Anbindung für poststationäre Nachsorgeuntersuchungen eingeleitet werden. Zur Versorgung von vaskulären Extremitätenverletzungen sollten sowohl endovaskuläre als auch offen-chirurgische Therapieoptionen vorgehalten werden. Ziel des Registers ist es, zur Evidenz bei der Versorgung von vaskulären Traumata beizutragen sowie Langzeitergebnisse für Rekonstruktionen nach vaskulärem Trauma zu erheben.

**Graphic abstract:**

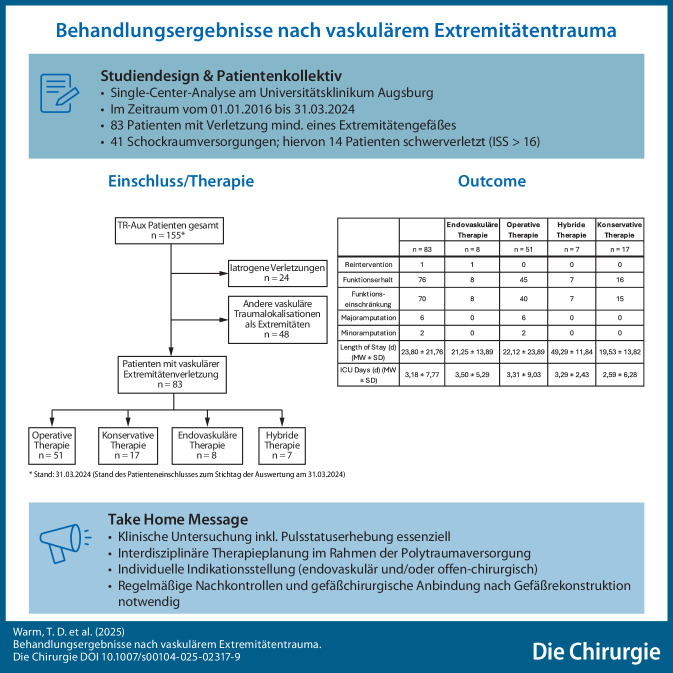

## Abstract (en)

**Background:**

A prospective vascular trauma registry (VascTR-Aux) is being conducted at Augsburg University Hospital. This first evaluation of the registry data aims to present the treatment results after vascular extremity injuries at discharge.

**Material and methods:**

From 1 January 2016 to 31 March 2024, a total of155 injured persons were included in the VascTR-Aux. Extremity vessels were affected in 83 patients.

**Results:**

Of the 83 injured people included, 62 were male. The average age was 37 years. The vascular injuries affected the upper limb in 28 cases and the lower limb in 55 cases. In 14 cases, the injuries were severe. The patients presented clinically with 29 hemorrhages, 32 ischemia and 14 combinations of both entities and 7 cases were asymptomatic. Patients were treated surgically (*n* = 51), as a hybrid procedure (*n* = 7) and as endovascular procedures (*n* = 8). In 76 cases functional preservation of the extremity was possible. Major amputation was necessary in six cases and minor amputation in two cases. The average length of stay was 23 days.

**Discussion:**

The initial treatment was often interdisciplinary, whereas inpatient follow-up care is often provided by specialist departments other than vascular surgery due to the concomitant injuries requiring treatment. The vascular surgery procedure for treatment and follow-up care must be discussed and communicated individually in the case of vascular injuries, and a vascular surgery follow-up protocol must be established for post-inpatient follow-up examinations. Both endovascular and open surgical treatment options should be available for the treatment of vascular limb injuries. The aim of the registry is to contribute to evidence for the treatment of vascular trauma and to collect long-term results for reconstructions after vascular trauma.

## Hintergrund

Traumatische Verletzungen mit vaskulärer Beteiligung sind seltene, jedoch oft schwerwiegende Verletzungen und stellen eine komplexe Herausforderung für das gesamte Traumateam in der interdisziplinären Behandlung dar [20].

Nach Auswertung von Datensätzen des deutschlandweiten Traumaregisters der Deutschen Gesellschaft für Orthopädie und Unfallchirurgie liegt bei mehrfach Schwerverletzten in bis zu 10 % der Fälle eine Gefäßbeteiligung vor. Die Mortalitätsrate bei Vorhandensein eines vaskulären Traumas wird mit 2–12 % angegeben [12].

Die Diagnostik und Behandlung von Gefäßverletzungen stellt daher eine absolute Notfallsituation dar. Die Therapie ist häufig komplex und erfordert fortgeschrittene operative oder interventionelle Techniken [6].

Da traumatische Gefäßverletzungen insgesamt selten sind, gestaltet es sich schwierig, bei der geringen Datenmenge valide Empfehlungen zu deren Behandlungen zu geben [10, 20].

Am Universitätsklinikum Augsburg wird aus diesen Gründen seit Januar 2016 prospektiv ein vaskuläres Traumaregister (VascTR-Aux) durch die Klinik für Gefäßchirurgie und endovaskuläre Chirurgie geführt.

Die vorliegende Arbeit umfasst die Auswertung von Behandlungsergebnissen nach vaskulärem Extremitätentrauma zum Entlasszeitpunkt.

## Patienten und Methoden

Die Erhebung wurde als Single-Center-Analyse am Universitätsklinikum Augsburg durchgeführt, welches als Haus der Maximalversorgung als Level-1-Traumacenter zertifiziert ist. Jährlich werden in diesem zwischen 800 und 1000 Schockraumversorgungen durchführt. Im Jahr 2021 beispielsweise wurden hiervon 355 Patienten in das TraumaNetzwerk der Deutschen Gesellschaft für Unfallchirurgie (DGU®) eingeschlossen; von diesen wurden 146 Patienten als schwerverletzt mit einem Injury Severity Score ≥ 16 (ISS) eingestuft.

**Abb. 1:**
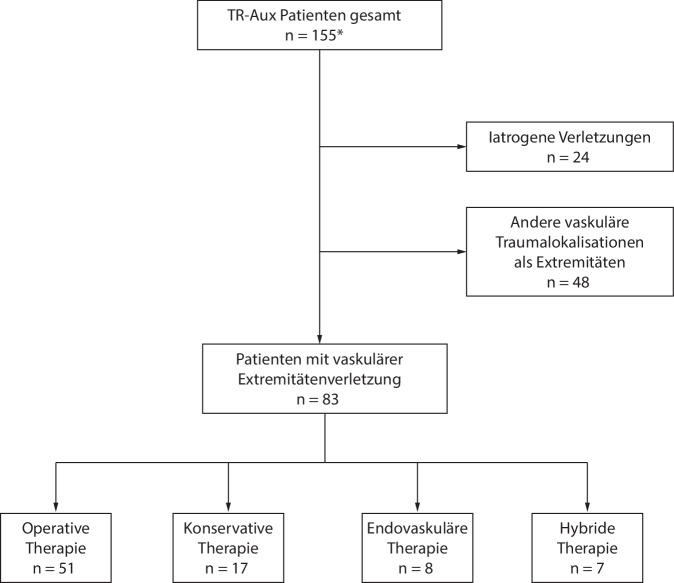
Grafische Darstellung der Patientenrekrutierung (*Sternchen*: Stand: 31.03.2024)

Vom 01.01.2016 bis 31.03.2024 wurden in das VascTR-Aux 155 Patienten mit traumatischer Gefäßverletzung prospektiv im Rahmen einer monozentrischen Beobachtung eingeschlossen. Davon war in 83 Fällen eine Extremität betroffen (Abb. 1).

Einschlusskriterien für die vorliegende Auswertung waren eine traumatisch bedingte Verletzung an arteriellen Leitgefäßen oder des tiefen Venensystems an der oberen und/oder unteren Extremität. Minderjährige Patientinnen und Patienten wurden nach schriftlicher Einwilligung der Erziehungsberechtigten zur Studienteilnahme in die Datenerhebung eingeschlossen.

Ausschlusskriterien waren eine Gefäßverletzung anderer Lokalisation; Junction-Verletzungen wurden für diese Erhebung ausgeschlossen.

Für die Einschätzung der Amputationswahrscheinlichkeit wurde der mehrfach validierte Mangled Extremity Severity Score (MESS) verwendet [2]. Als schwerverletzt wurden alle Patienten mit einem ISS ≥ 16 gezählt [4]. Eine präoperative Risikoeinschätzung erfolgte nach der Klassifikation der American Society of Anesthesiologists (ASA) [1].

Bezüglich der Einteilung nach dem Mechanismus des Traumas wurde ein Hochrasanztrauma als ein Unfall mit einer Geschwindigkeit ≥ 100 km/h oder einer Geschwindigkeitsänderung (∆V) ≥ 30 km/h definiert. Ein Sturz aus großer Höhe wurde als eine Sturzhöhe ≥ 3 m bestimmt gemäß der gültigen S3-Leitlinie Polytraumversorgung [4].

Die Datenerfassung erfolgte mittels Microsoft-Excel® (Version 16.43; Microsoft Corporation, Redmond, WA, USA).

Es wurden deskriptive Statistiken erstellt. Die stetigen Daten wurden mit dem Kruskal-Wallis-Test und Mann-Whitney-U-Test für unabhängige Stichproben auf Signifikanz untersucht. Die kategorialen Daten wurden mit dem exakten Test nach Fisher auf Signifikanz untersucht. Als Auswertungsprogramm diente SPSS (Version 29.0; IBM Corp., Armonk, NY, USA).

Die Führung des Registers wurde von der Ethikkommission bei der Ludwig-Maximilian-Universität München genehmigt (Projekt-Nr.: 23-0312). Eine Registrierung bei Clinical Trials ist erfolgt (Nr.: NCT05846321).

## Ergebnisse

Von den 83 eingeschlossenen Teilnehmenden waren 62 männlich und 21 weiblich. Im Median betrug das Alter 37 Jahre (min./max. 4 bis 80 Jahre).

Eine Übersicht über die Vorerkrankungen, den ASA-Status sowie die klinische Präsentation der Gefäßverletzungen gibt Tab. 1.

**Tab. 1:** Patientencharakteristika

		Endovaskuläre Therapie	Operative Therapie	Hybride Therapie	Konservative Therapie	*p*-Wert
	*n* = 83	*n* = 8	*n* = 51	*n* = 7	*n* = 17	
Geschlecht (m/w/d)	62/21/0	6/2/0	39/12/0	6/1/0	11/6/0	–
Alter (y) MW ± SD	37,41 ± 21,17	40,00 ± 27,15	37,51 ± 20,65	37,00 ± 18,42	36,06 ± 22,59	0,10
Medianes Alter (y)	37,00	30,50	38,00	30,00	42,00	0,87
Vorerkrankungen						
BMI > 30 kg/m^2^	14	4	5	1	4	0,78
aHT	14	2	8	3	1	0,13
HLP	3	0	3	0	0	0,76
Nikotin	12	1	7	1	3	0,95
DM II	2	0	2	0	0	1,00
ASA-Klassifikation						
1	32	4	19	1	8	0,46
2	32	4	21	1	6	0,53
3	10	0	6	3	1	0,77
4	9	0	5	2	2	0,38
5	0	0	0	0	0	–
6	0	0	0	0	0	–
Klinische Präsentation						
Ischämie	32	7	12	4	9	< 0,001
Blutung	29	1	24	1	3	0,04
Blutung und Ischämie	14	0	12	2	0	0,04
Asymptomatisch	7	0	3	0	4	0,14
Schockraumversorgung	41	4	23	14	4	0,76
Schwerverletzt (ISS > 16)	14	2	7	1	10	0,68
Verletzte Gefäßregion						
Obere Extremität	28	2	18	1	5	–
Rechte obere Extremität	9	0	7	1	1	0,67
Linke obere Extremität	19	2	13	0	4	0,62
Untere Extremität	55	6	32	6	12	–
Rechte untere Extremität	24	2	15	1	6	0,86
Linke untere Extremität	31	4	16	5	6	0,19
Anzahl der verletzten Gefäße						
Isoliert 1 Gefäß verletzt	70	6	44	5	15	0,53
Mind. 2 Gefäße verletzt	13	2	7	2	2	0,53

*BMI* Body-Mass-Index (kg/m^2^), *aHT* arterielle Hypertonie, *HLP* Hyperlipidämie, *DM II* Diabetes mellitus Typ 2, *ASA* American Society of Anesthesiologists, *ISS* Injury Severity Score

Es lag in 55 Fällen eine Gefäßverletzung an der unteren und in 28 Fällen an der oberen Extremität vor.

**Abb. 2:**
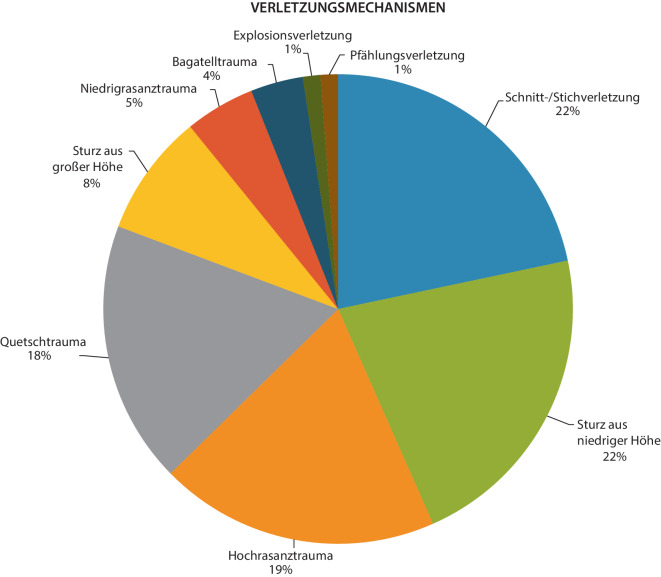
Eingeschlossene Verletzungsmechanismen

Die Verletzungsmechanismen sind in Abb. 2 dargestellt.

Die initiale Bildgebung war in der Mehrzahl der Fälle die CT-Angiographie, weitere Optionen zeigt Tab. 2.

**Tab. 2:** Diagnostische Maßnahmen bei Aufnahme (*n* = 83)

Bildgebende Diagnostik*	
CT-Angiographie	56
FKDS	7
DSA	13
Direkte klinische Exploration ohne vorherige Bildgebung	15
Pulsstatus an der Extremität bei Aufnahme	
Tastbar	21
Nicht tastbar	52
Nicht dokumentiert	10

^*^Mehrfachnennung möglich

Die peripheren Pulse an der betroffenen Extremität waren bei Aufnahme in 52 Fällen nicht tastbar, in 21 Fällen tastbar, und 10-mal erfolgte keine Dokumentation des initialen Pulsstatus.

Die offen chirurgische Versorgung erfolgte in 51 Fällen, in 8 Fällen wurde eine rein endovaskuläre Therapie angewendet, in 7 Fällen erfolgte eine kombiniert endovaskuläre und offen chirurgische Therapie (Hybridtherapie), und 17-mal wurde eine rein konservative Therapie durchgeführt (Tab. 3).

**Tab. 3:** Outcome

		Endovaskuläre Therapie	Operative Therapie	Hybride Therapie	Konservative Therapie	*p*-Wert
	*n* = 83	*n* = 8	*n* = 51	*n* = 7	*n* = 17	
Reintervention	1	1	0	0	16	0,18
Funktionserhalt	76	8	45	7	15	0,85
Funktionseinschränkung	70	8	40	7	0	0,37
Majoramputation	6	0	6	0	0	0,42
Minoramputation	2	0	2	0	–	1,00
Length of Stay (d)						
MW ± SD	23,80 ± 21,76	21,25 ± 13,89	22,12 ± 23,89	49,29 ± 11,84	19,53 ± 13,82	0,007
Median	16,00	16,50	13,00	53,00	19,00	–
ICU Days (d)						
MW ± SD	3,18 ± 7,77	3,50 ± 5,29	3,31 ± 9,03	3,29 ± 2,43	2,59 ± 6,28	–
[Min.; Max.]	[0; 45]	[0; 13]	[0; 45]	[0; 6]	[0; 21]	0,24
Entlassung						
Nach Hause	71	6	46	4	15	0,08
In eine AHB	10	1	4	3	2	0,06
In ein anderes KH	2	1	1	0	0	0,37
ABI bei Entlassung						
Dokumentiert	38	5	22	3	8	0,81
Nicht dokumentiert	45	3	29	4	9	0,81

*AHB* Anschlussheilbehandlung, *KH* Krankenhaus, *AIN* Anästhesie und Intensivmedizin, *GCH* Gefäßchirurgie und endovaskuläre Chirurgie, *NCH* Neurochirurgie, *TCH* Thoraxchirurgie, *KCH* Kinderchirurgie, *UCH* Unfallchirurgie, Orthopädie und Handchirurgie, *ABI* Ankle-Brachialis-Index

In einem Fall war eine Reintervention während des stationären Aufenthaltes notwendig.

In 76 Fällen war ein Funktionserhalt der betroffenen Extremität möglich. In 6 Fällen musste eine Majoramputation der Extremität erfolgen. Bei einem weiteren Patienten wurde im Verlauf eine Majoramputation indiziert; der Patient wünschte stattdessen eine Verlegung in eine andere Klinik zur Zweitmeinung. Die Indikationen für die durchgeführten Majoramputationen ergaben sich aus der Komplexität der Verletzungen mit ausgeprägtem Schaden an Weichteilen, knöchernen Strukturen und/oder Nervengewebe. Ein MESS ≥ 7 lag bei allen durchgeführten primären und sekundären Majoramputationen vor.

Weiterhin wurde bei 2 Patienten primär eine Minoramputation an der verletzten Extremität aufgrund des bestehenden traumatischen Gewebeschadens durchgeführt.

Die Krankenhausverweildauer lag im Median bei 16 Tagen (MW ± SD: 23,80 ± 21,76), der Aufenthalt auf ICU bei 3 Tagen (MW ± SD: 3,18 ± 7,77).

Die Entlassung aus der stationären Behandlung erfolgte durch folgende Fachabteilungen: in 52 Fällen durch die Klinik für Unfallchirurgie und Orthopädie, in 17 Fällen durch die Klinik für Gefäßchirurgie und endovaskuläre Chirurgie, in 10 Fällen durch die Klinik für Kinderchirurgie und in 2 Fällen direkt durch die Klinik für Anästhesiologie und operative Intensivmedizin direkt von der operativen Intensivstation und in jeweils einem Fall durch die Kliniken für Neuro- und Thoraxchirurgie.

## Diskussion

### Patientenkollektiv

Die erhobenen Daten zeigen, dass es sich bei den Verunfallten, um junge und gefäßgesunde Patientinnen und Patienten handelt (s. Tab. 1). Überwiegend handelt es sich um Männer zwischen 30 und 40 Jahren; dies ist übereinstimmend mit anderen Publikationen, die ein ähnliches Patientenkollektiv im zivilen Setting beschreiben [8, 14]. In 20 (24,1 %) Fällen war die verunfallte Person noch nicht volljährig. Dabei lag 11-mal eine isolierte Gefäßverletzung an der oberen Extremität vor, meist im Zusammenhang mit einer suprakondylären Humerusfraktur, bei welcher initial oder nach Reposition ein Pulsdefizit oder klinische Ischämiezeichen vorlagen. Dabei ist eine Verletzung der A. brachialis bei der suprakondylären Humerusfraktur mit einer Inzidenz von bis zu 19 % als eine der häufigsten Gefäßverletzungen bei Kindern beschrieben [16].

Die klassischen kardiovaskulären Risikofaktoren lagen in diesem Patientenkollektiv selten vor.

In der Erhebung war in der Mehrzahl der Fälle die untere Extremität (66 %) betroffen. In der Literatur wird ein Verhältnis von Gefäßverletzungen der oberen und unteren Extremität von 30 zu 70 % angegeben [7, 21].

#### Diagnostik

Als primäre Diagnostik hat sich die Durchführung der CT-Angiographie bei vaskulären Verletzungen etabliert und wird auch durch die Fachgesellschaften insbesondere bei polytraumatisierten Patienten empfohlen [4, 20].

Alternativ kann bei ausgewählten Verletzungsmustern und bei kreislaufstabilen Patienten eine digitale Subtraktionsangiographie (DSA) und ggf. eine sich direkt anschließende interventionelle Therapie erfolgen.

Trotz der flächendeckenden Etablierung der CT-Angiographie darf gerade bei dem Verdacht auf vaskuläre Begleitverletzungen die klinische Dokumentation der Durchblutung und Sensomotorik der Extremität (pDMS) bei Erstvorstellung nicht unterlassen werden.

Alternativ zur Computertomographie kann bei dem Verdacht auf eine isolierte traumatische Gefäßverletzung, welche in der Mehrzahl der von uns erhobenen Fälle vorlag, die farbkodierte Duplexsonographie als nichtinvasive und flächendeckend verfügbare Diagnostik erfolgen. Einschränkungen für die FKDS bestehen bei peripheren Gefäßverletzungen und erschwerter Zugänglichkeit durch Begleitverletzungen oder bereits angelegten medizinischen Hilfsmitteln wie beispielsweise Frakturschienen. Bei isolierten peripheren Gefäßverletzungen an den Extremitäten kann in einigen Fällen, beispielsweise bei lokalisierten Schnittverletzungen, eine direkte operative Exploration erfolgen und auf eine weitere präoperative Diagnostik verzichtet werden.

#### Therapie

Die erhobenen Daten zeigen, dass die initiale Therapie von Traumata mit vaskulärer Beteiligung häufig interdisziplinär erfolgt, oftmals aufgrund der Verletzungsschwere und/oder bestehender Begleitverletzungen im Rahmen der interdisziplinären Schockraumversorgung. Auch in der gültigen deutschen S3-Leitlinie zur Polytraumaversorgung und der im Januar 2025 neu publizierten Leitlinie zur Versorgung von vaskulären Traumata der European Society for Vascular Surgery (ESVS) wird die Versorgung von Gefäßverletzungen der Extremitäten so früh wie möglich empfohlen [4, 20].

Es zeigte sich, dass trotz der zunehmenden endovaskulären Therapieoptionen die Mehrzahl der traumatischen Gefäßverletzungen primär offen gefäßchirurgisch versorgt wurde.

Grundsätzlich kommt bei traumatischen Gefäßverletzungen das gesamte Spektrum der offen-gefäßchirurgischen Therapie zum Einsatz; am häufigsten erfolgten die Anlage eines Interponats und die direkte Gefäßnaht.

Die vorliegende Erhebung zeigt, dass auch in der Versorgung traumatisierter Patienten die endovaskuläre Therapie ihren Stellenwert hat und interventionelle Techniken zur Blutungskontrolle und Rekanalisation eingesetzt werden.

Offene und interventionelle Methoden sollten nicht als konkurrierende Verfahren angesehen werden, sondern als sich ergänzende Möglichkeiten der Versorgung. Die Kriterien zur Patientenselektion ergeben sich aus Verletzungsmuster und -ausdehnung (z. B. lokale Dissektion, nichtzirkuläre Verletzungen, Blutungen im peripheren Stromgebiet), anatomischer Höhe der Läsion (z. B. Lage außerhalb eines Bewegungssegments) sowie Kreislaufstabilität des Verletzten.

In der gültigen S3-Leitlinie wird empfohlen, wenn möglich auf die Anwendung endovaskulärer Techniken aufgrund ihrer geringeren Invasivität und des möglichen Zeitvorteils zur Blutungskontrolle zurückzugreifen [4].

Bei vorhandener Infrastruktur vor Ort sollte gerade bei Schwerverletzten die simultane Durchführung von Diagnostik und Therapie mit Angiographie und endovaskulärer Therapie aufgrund des geringeren Zugangstraumas und des zeitlichen Benefits für den Patienten in einem Hybridoperationssaal favorisiert werden [18].

In unserer Erhebung erfolgte die Versorgung im Hybridoperationssaal in 7 Fällen. Zwei Prozeduren stellten eine Kombination aus initialer Blutungskontrolle mittels Blockadeballon und anschließender offen-chirurgischer Versorgung dar. In weiteren 2 Fällen konnte nach frustranem Rekanalisationsversuch direkt die operative Revaskularisation bzw. Blutstillung erfolgen.

Nach den oben genannten Therapieformen wurde eine zweizeitige Kompartmentspaltung in 2 Fällen bei offen-chirurgischer, in 3 Fällen bei endovaskulärer und in 2 Fällen bei Hybridversorgung durchgeführt. Zweizeitige Majoramputationen waren in 4 Fällen bei der rein offen-chirurgischen Versorgung notwendig, nie bei endovaskulärer oder hybrider Therapie. Zweizeitige Minoramputationen waren in keinem Fall notwendig.

Die Abwägung bezüglich einer offen-chirurgischen, einer endovaskulären, hybriden oder rein konservativen Therapie sollte immer individuell abhängig vom Zustand des Patienten und der lokalen Infrastruktur des Traumazentrums getroffen werden. Es konnte kein signifikanter Unterschied zwischen den Eingriffsdauern der endovaskulären, chirurgischen und hybriden Therapieverfahren aufgezeigt werden. In unserem Kollektiv zeigte sich zwar eine deutlich längere Eingriffsdauer bei Anwendung von hybriden Therapieverfahren, jedoch handelte es sich in diesen Fällen um komplexe oder kombinierte Therapieverfahren.

#### Therapieergebnisse

Erfreulicherweise war die Rate an Majoramputationen bei unserer Erhebung mit insgesamt 6 Amputationen (7,2 %) niedrig. In der Literatur werden mit bis zu 20 % deutlich höhere Zahlen beschrieben [8, 13]. Größere aktuelle Studienkollektive fehlen jedoch, und neuere endovaskuläre Therapieoptionen lagen bei den damaligen Erhebungen möglicherweise noch nicht vor. Die zweizeitig erfolgten Amputationen unseres Kollektivs waren im stationären Verlauf in allen Fällen aufgrund des ausgedehnten Weichteilschadens und bestehender Begleitverletzungen der Extremität notwendig und in keinem Fall vaskulär bedingt.

Eine Erklärung für die vorliegende niedrige Amputationsrate sehen wir in der primären multidisziplinären Behandlung von vaskulären Extremitätentraumen in einem Level-1-Traumacenter. Dabei erfolgt in der Regel zunächst die primäre Frakturversorgung und im direkten Anschluss die vaskuläre Ausversorgung. Eine Ausnahme stellt hier eine starke oder unstillbare Blutung dar, welche eine primäre Blutstillung, beispielsweise durch endovaskuläre Ballonblockade notwendig macht. Anders als im militärischen Setting wird die Anlage von temporären Shunts zur Revaskularisation im zivilen Setting nicht empfohlen, sofern eine zeitnahe definitive Ausversorgung möglich ist [20]. Ein REBOA-Manöver sehen wir bei peripheren Extremitätentraumen nicht indiziert, es kann jedoch bei den stammnahen Verletzungen zum Einsatz kommen.

Weitere mögliche Faktoren für das gute primäre Outcome sind die Vorhaltung aller endovaskulären und offen-chirurgischen Therapieoptionen sowie die Schulung der Operateure in vaskulären traumatologischen Techniken. Eine endovaskuläre Grundausstattung stellen hierbei Blockadeballone zur temporären Blutstillung, Stentgrafts zur Blutstillung und Coils zum Verschluss von blutenden Seitenästen dar. Das Festlegen eines Algorithmus für Diagnostik und Therapie erleichtert im Akutfall die Entscheidungsfindung. Unser Algorithmus für die iatrogene Verletzung der A. poplitea wurde bereits veröffentlicht [9].

Obgleich bei den erhaltenen Extremitäten im Studienkollektiv eine hohe generelle Funktionsfähigkeit (90,4 %) bestand, wies die Mehrzahl (84,3 %) der Patienten bei Entlassung aus der stationären Therapie noch Funktionseinschränkungen an der betroffenen Extremität auf. Diese waren in allen Fällen durch die knöchernen, bindegewebigen und nervalen Verletzungen bedingt, sodass eine Vollbelastung aus unfallchirurgischer Sicht noch nicht möglich war.

Die Reinterventionsrate nach Gefäßtrauma lag in unserem Kollektiv bei 1,2 % (*n* = 1). Die Reintervention in unserer Erhebung trat nach endovaskulärer Primärtherapie auf: Nach einer endovaskulären Versorgung mittels Stentgraft war bei einem Frühverschluss nach 7 Tagen eine Revision mittels Aspirationsthrombektomie notwendig.

Eine niedrige Reinterventionsrate von arteriellen Gefäßrekonstruktionen bei traumatisierten Patienten wird beispielsweise auch von Klocker et al. über einen Nachbeobachtungszeitraum von bis zu 5 Jahren berichtet [15]. Langzeitdaten in ausreichender Anzahl für Offenheitsraten von Gefäßrekonstruktionen liegen für vaskuläre Traumata bisher nicht vor.

Die gerinnungshemmende Therapie erfolgte bei dem untersuchten Patientenkollektiv in der Frühphase nach Operation analog zu etablierten Therapieschemata nach Revaskularisation bei gefäßchirurgischen Patienten mit einer pAVK [3]. Individuell angepasst wurden diese medikamentösen Therapieregime abhängig von den bestehenden traumatischen Begleitverletzungen.

Zur Weiterbehandlung mit gerinnungshemmenden Medikamenten nach Gefäßtrauma liegen aktuell nur wenige Erfahrungsberichte mit geringer Probandenzahl und kurzem Nachbeobachtungszeitraum vor [11, 15, 17].

Es bestehen Hinweise darauf, dass die Offenheitsraten der Gefäßrekonstruktionen besser sind als bei Patienten mit arteriosklerotischen Gefäßpathologien (pAVK) und die medikamentöse Gerinnungshemmung eine geringere Rolle spielt. So zeigte das PROspective Observational Vascular Injury Trial (PROOVIT) Register der American Association for the Surgery of Trauma (AAST), dass im primären stationären Aufenthalt kein Unterschied in der Offenheitsrate von Interponaten unabhängig von der gerinnungshemmenden Medikation vorliegt [5]. In unserem Zentrum erhalten die Patienten initial eine Gerinnungshemmung nach Empfehlungen bei Patienten mit pAVK, ggf. mit kürzerer Dauer nach klinischer Reevaluation in der Regel nach 6 Monaten.

Da die stationäre Weiterbetreuung der Verletzten aufgrund von behandlungsbedürftigen Begleitverletzungen häufig in anderen Fachabteilung als der Gefäßchirurgie erfolgt, sollte das spezifische Procedere frühzeitig dokumentiert werden. Hierzu gehören die Dokumentation des vaskulären Status und die Einleitung einer poststationären gefäßmedizinischen Anbindung.

#### Nachsorge

Alle traumaassoziierten Gefäßverletzungen, auch solche, welche rein konservativ behandelt werden, sollen aufgrund der möglichen Entstehung von Langzeitfolgen wie Stenosebildung oder Aneurysmaentwicklung in regelmäßigen Abständen gefäßmedizinisch nachkontrolliert werden, um mögliche Spätfolgen frühzeitig zu erkennen [19, 20].

Im Unterschied zu den gefäßchirurgischen Patienten mit chronischen Gefäßerkrankungen ist das hier vorliegende Patientenklientel deutlich jünger und weist weniger Vorerkrankungen und kardiovaskuläre Risikofaktoren auf. Daher können die Ergebnisse aus der gefäßchirurgischen Forschung mit vaskulärer Grunderkrankung nur eingeschränkt übertragen werden. Leitlinienempfehlungen zu Therapie, Antikoagulation oder Nachsorgeschemata existieren bisher nicht.

#### Ausblick

Der Mangel an Evidenz bezüglich des mittel- bis langfristigen Follow-up von traumaassoziierten Gefäßverletzungen wird auch in der Leitlinie zum Management von traumatischen Gefäßverletzungen der ESVS aufgezeigt und stellt das nächste Projekt des VascTR-Aux dar. Aufgrund der geringen Fallzahlen von traumatischen Gefäßverletzungen im zivilen Setting ist perspektivisch eine multizentrische Datenerhebung, beispielsweise in einem deutschlandweiten vaskulären Traumaregister, für die Schaffung ausreichender Evidenz wünschenswert.

## Fazit für die Praxis

- Bei Verdacht auf Gefäßverletzung ist die klinische Untersuchung der betroffenen Extremität mit Erhebung des Pulsstatus essenziell.
- Als primäre Diagnostik ist die Durchführung einer CT-Angiographie bei Traumapatienten empfohlen.
- Die Therapieplanung und Durchführung sollen im Rahmen einer Schockraumversorgung interdisziplinär erfolgen.
- Als Therapieoptionen bieten sich sowohl offen-chirurgische als auch endovaskuläre gefäßrekonstruktive Techniken an. Diese sollen je nach Verletzungsmechanismus individuell diskutiert werden.
- Regelmäßige Nachkontrollen nach Gefäßtraumen sind notwendig, um Komplikationen frühzeitig zu erkennen und ggf. behandeln zu können. Standardisierte Schemata müssen hierzu mit Evidenz erarbeitet werden.
- Langzeitergebnisse nach Gefäßrekonstruktion bei traumassoziierten Gefäßverletzungen sind notwendig, um Antikoagulations- und Nachsorgeschemata festzulegen.

## Data Availability

Die erhobenen Datensätze können auf begründete Anfrage in anonymisierter Form beim korrespondierenden Autor angefordert werden. Die Daten befinden sich auf einem Datenspeicher der Klinik für endovaskuläre Chirurgie und Gefäßchirurgie des Universitätsklinikums Augsburg.
